# An online expert network for high quality information on occupational safety and health: cross-sectional study of user satisfaction and impact

**DOI:** 10.1186/1472-6947-11-72

**Published:** 2011-11-23

**Authors:** Martijn DF Rhebergen, Annet F Lenderink, Frank JH van Dijk, Carel TJ Hulshof

**Affiliations:** 1 Coronel Institute of Occupational Health, Academic Medical Center Amsterdam/University of Amsterdam, PO Box: 22700, 1100 DE Amsterdam, The Netherlands; 2 Netherlands Center for Occupational Diseases, Academic Medical Center Amsterdam/University of Amsterdam, PO Box: 22660, 1100 DD, Amsterdam, The Netherlands; 3 Centre of Excellence, Netherlands Society of Occupational Medicine (NVAB), PO Box 2113, 3500 GC Utrecht, The Netherlands

## Abstract

**Background:**

Many people have difficulties finding information on health questions, including occupational safety and health (OSH) issues. One solution to alleviate these difficulties could be to offer questioners free-of-charge, online access to a network of OSH experts who provide tailored, high-quality information. The aim of this study was to assess whether network quality, respectively information quality, as perceived by the questioners, is associated with questioners' overall satisfaction and to explore the impact of the information received on questioners' knowledge, work and work functioning.

**Methods:**

We evaluated the experiences of OSH questioners with the online network ArboAntwoord.com over a two-year period. In this network, approximately 80 qualified experts are available to answer OSH questions. By means of a questionnaire, we assessed questioners' overall satisfaction with the network, whether the network was user-friendly, easily accessible and easy to handle and whether the information provided was complete, applicable and received in a timely manner. The impact of the information on questioners' knowledge, work or work functioning was explored with seven questions. In the study period, 460 unique OSH questioners asked 851 OSH questions. In total, 205 of the 460 questioners completed the questionnaire (response rate 45%).

**Results:**

Of the responders, 71% were satisfied with the ArboAntwoord network. Multiple logistic regression analysis showed that the applicability of the information had a positive influence on the questioners' overall satisfaction (OR = 16.0, 95% CI: 7.0-36.4). Also, user friendliness of the network (OR = 3.3, 95% CI: 1.3-8.6) and completeness of the information provided (OR = 3.0, 95% CI: 1.3-6.8) were positively related to the questioners' satisfaction. For 74% of the questioners, the information helped to increase their knowledge and understanding. Overall, 25% of the questioners indicated that the received information improved their work, work functioning or health.

**Conclusions:**

A free-of-charge, online expert network in the field of OSH can be a useful strategy to provide OSH questioners with applicable, complete and timely information that may help improve safety and health at work. This study provides more insight in how to satisfy network questioners and about the potential impact of provided information on OSH.

## Background

Many workers, managers and occupational safety and health (OSH) professionals seek information on OSH risks, interventions and legislation [1-5]. Easy access to high-quality OSH information and advice may empower information seekers not only by increasing knowledge and understanding but also by improving how they cope with topics of work and health, such as improving poor working conditions [6-10]. However, many individuals have difficulties in finding high-quality OSH information [3,11,12].

Finding high-quality health information often requires specific skills or "health literacy" [13,14]. The World Health Organisation defines health literacy as "the cognitive and social skills which determine the motivation and the ability of an individual to gain access to, understand and use information in ways that promote and maintain good health" [15]. Both promoting health literacy by education and improving access to quality information could support OSH questioners in their search for answers.

Most workers, managers and OSH professionals access the Internet, or ask advice from OSH experts to find quality information [2,3,11,16-18]. Although easily accessible, the internet often provides excessive amounts of information, which is not always of high quality [19-23]. Well-trained experts might be better able to provide questioners with tailored information or advice of high quality [24,25]; however consulting trained experts may be hampered by restricted access and high costs [11,26]. One solution might be to offer OSH questioners the ability to approach experts by computer or telephone free-of-charge [27-32].

For this purpose, several countries have set up telephonic or online expert services that provide information and advice to OSH questioners [1,4,5,16,31]. An online expert network has the additional advantage of providing easy access to many experts [31,33]. Online expert networks allow knowledge to be stored and re-used more easily than telephonic helpdesks. Therefore, this knowledge will not be lost for the organisation or other questioners. Online networks can be accessed by computer or (smart)phone. Recently, Google and Yahoo launched similar online networks [34,35]. We launched the online expert network ArboAntwoord, which is meant for OSH questioners, in October 2008 by a small scale campaign [31]http://www.arboantwoord.com.

Knowledge about the satisfaction of questioners and the impact of information and advice provided might help to create or redesign health-related expert services. Although a number of expert services have been shown to result in satisfied questioners [4,36,37], it is still unknown which factors are associated with questioners' satisfaction and what the impact of the provided information and advice is. In other studies, perception of the quality of a new information facility (including user friendliness and accessibility) and the quality of the information provided (including information completeness and applicability) have been associated with high user satisfaction [38-40].

Accordingly, the aim of this study is to assess whether, and to what extent, perceived network quality and perceived information quality are associated with overall satisfaction with the online expert network ArboAntwoord. The information and advice provided by experts in ArboAntwoord may improve questioners' knowledge and understanding, and consequently their work or work functioning. Exploring the impact of information on OSH questioners is the second aim of this study.

## Methods

### Study design and participants

In a two-year study period, from May 1, 2009, until May 1, 2011, we evaluated the experiences of OSH questioners of the online network ArboAntwoord by means of a periodic questionnaire.

#### ArboAntwoord

The aim of the ArboAntwoord expert network is to answer OSH questions free of charge, within one week. In ArboAntwoord, individuals can pose their question directly to one or more experts whose expertise matches their needs. The network facilitates the interaction by sending an e-mail notification to the selected expert (when questioned) and to the questioner (when answered). Questions are occasionally answered by more than one expert. Eligible questions and answers are stored in a searchable database for public use after moderation and anonymisation only if informed consent by the questioner is obtained. The development and features of the ArboAntwoord network are described in detail in another paper [31].

The primary target audience is the working population with OSH questions, including employees, employers, (line) managers and human resource professionals (HRM). The network could also be used by OSH professionals such as occupational physicians and "OSH supervisors" (in Dutch: preventiemedewerkers). Initially, approximately 70 Dutch OSH experts were invited to participate in the network if they met all the following criteria: 1) working in a university, expert centre, or highly qualified OSH organisation operating on a national level, 2) having (inter)national expertise on a specific OSH topic, 3) having at least five years of experience on this topic and 4) participating in at least one knowledge dissemination activity such as authorship of scientific articles or participation on an expert committee. During the study period several new experts were invited to participate, while several others quit for various reasons (e.g., migration, change of job). Currently, a steady network of about 80 experts participate in the network encompassing a wide variety of OSH professions, such as occupational physicians, hygienists, safety engineers, human movement scientists, health scientists, (neuro) psychologists, dermatologists, internists, lawyers and OSH legislation specialists.

In the two-year study period, ArboAntwoord received 62,000 unique visitors (Google Analytics). In total, 851 questions were received from 460 different questioners, an average of 1.9 questions per questioner. During the study period, 37% of the questions were published online, and on average, each question was viewed 337 times by other information seekers. Approximately 40% of the questions were related to OSH laws and regulation, e.g., "Are safety shoes obligatory for workers in an army storage depot?" Sixteen percent of the questions focused on interventions to improve working conditions or on treatment for an occupational disease, e.g., "What is an effective intervention to prevent work-related hearing loss for physical education teachers?" Thirteen percent related to causes of and risk factors for work-related health and safety problems, e.g., "Is noise a serious risk for physical education teachers?" On average, experts answered the questions within 2.2 days. In 90% of cases, experts needed less than 30 minutes to answer a question.

### Data collection

For this study, we defined four outcomes: 1) the perceived quality of the online network ArboAntwoord facility, 2) the perceived quality of the information provided by experts, 3) the questioner's overall satisfaction with the network services and 4) the impact of the provided information on the questioner. We transformed these outcomes into questions in a web-based questionnaire. The questions were directly based on questions from previous studies on the success of (new) information systems, especially studies concerning the well-known model of information systems success developed by DeLone & McLean [38-40]. Questions found in the literature were translated and adjusted to fit the purpose of our study. Three questions were related to the perceived "technical" quality of the network. We asked if the network was user friendly, easily accessible, and easy to handle. These variables have all been shown to be relevant to the perceived quality of a new information facility [38-40]. To assess the perceived quality of the actual information provided, we formulated three questions related to completeness, applicability and timeliness of the information received. These variables have been shown to be important to the quality of information that a facility provides [38-40]. One single item question was dedicated to the overall satisfaction of the questioner with the network as an information facility, i.e., "Overall, how satisfied are you with ArboAntwoord as an information facility?" The questions were answered on five-point Likert-scales (completely disagree-completely agree).

We decided to distinguish between the impact of information on the questioners' knowledge and understanding and the impact on their work or work functioning [41]. To assess the impact on the questioners' knowledge and understanding, we formulated three questions (five point Likert-scales; completely disagree-completely agree; 0-4 points): "Did the answer of the expert(s) bring you closer to the solution of your question or situation?", "Did the answer increase your knowledge or understanding of the situation?" and "Did the answer provide you with the opportunity to exclude other potential answers?" In addition, we asked "Can you please clarify your response to these three questions (open-ended)?" To explore the impact of information on questioners' work and work functioning we formulated three questions: "Did or do you have the intention to actually apply the answer to your work situation (not applicable, no, yes)?", "Did the answer affect, or will the answer affect your work or work functioning (yes or no)?" and "If yes, what did or will it affect (open-ended)?" We decided not to ask a specific question on prevention or recovery of illness or accidents because this type of impact might be difficult for questioners to estimate, and effects may only be realised after several years. To collect data on background characteristics of the questioners, we included questions on gender, age, educational level, occupation, company size, company sector and internet use. Background data from the questioners' web domains, collected during their registration to the ArboAntwoord website, was used as an additional source of information on non-responders.

The web-based questionnaire was tested for comprehensibility by four workers from different departments of the Academic Medical Center (AMC) in Amsterdam. By convenience sampling, we recruited one worker from the catering service, one from the children's day care centre, one nurse and one researcher. First, two workers were interviewed and their suggestions were included in the next two interviews and in the next version of the questionnaire. The final version of the questionnaire was sent to all individuals who posed a question to an expert in the ArboAntwoord network in the study period (N = 491). Questionnaires were sent periodically to all questioners in the preceding 90 days. We considered this period long enough to observe a potential impact of an answer and short enough to limit recall bias. A disadvantage to this approach is that the time between receiving an answer and the time to realise an impact varies between questioners from one to 90 days. Twenty-three questioners filled in more than one questionnaire (31 questionnaires). Only the first questionnaire was used for analysis (N = 460). It took questioners about 5 to 10 minutes to complete the questionnaire. Since this questionnaire study is not subject to the Dutch Medical Research Involving Human Subjects Act (WMO), approval of the Medical Ethical Committee was not required. Informed consent was obtained when a participant started the questionnaire.

### Data analysis

All outcomes were first analysed by means of descriptive analyses. All analyses were carried out using SPSS 17.0 (SPSS Inc., Chicago, IL, USA).

To evaluate which background, network quality or information quality variables were associated with the questioners' overall satisfaction we used the Chi-Squared test for dichotomous variables and the Yates & Cochrane test for ordinal variables (p < 0.05). For this analysis, scores of all separate variables of network quality, information quality, and questioners' overall satisfaction were dichotomised (0-2 = disagree; 3,4 = agree). The internal consistency of the three variables referring to the perceived network quality, respectively the perceived information quality, was poor (Cronbach's alpha < 0.6).

After checking for multicollinearity (Tolerance > 0.10), all significant predictors (p < 0.10) were included in a stepwise logistic regression model (backwards procedure; Likelihood Ratio). The Hosmer and Lemeshow test was used to establish goodness of fit (p > 0.05). For the stepping method we used as criteria p < 0.05 for entry and p > 0.05 for removal.

The internal consistency of the three variables (questions) referring to the impact of information received on questioners' knowledge and understanding was good (Cronbach's alpha = 0.86). Therefore, the average scores of these three questions were processed into one single-item variable, representing the impact of information on questioners' knowledge and understanding (0-4 points). Subsequently, this variable was dichotomised by using a cut-off score of 2.33 (< 2.33 = no impact on knowledge and understanding; ≥ 2.33 = impact on knowledge and understanding).

Finally, MR and CH performed a basic thematic content analysis on the remarks from the open-ended impact question. Discussions between MR and CH led to a simple taxonomy of only one domain for the question on knowledge and understanding and three domains for the question on work and work functioning. To evaluate which background variables were associated with impact on work or work functioning we used the Chi-Squared test for dichotomous variables and the Yates & Cochrane test for ordinal variables (p < 0.05).

## Results

### Questioner characteristics

Overall, 205 of the 460 questioners completed the questionnaire, a response rate of 45%. There were slightly more male than female responders and most were above the age of 39 years. Many had a high or intermediate educational level, about half used the internet between zero and ten 10 hours a week. The jobs of the questioners were widely spread over various sectors and sizes of companies (Table 1). In comparison to the ArboAntwoord questioners in general (web domain data), questioners between 40 and 59 years old, questioners with a low educational level and questioners working for a large company are slightly overrepresented in the group of respondents. Young questioners between 15 and 29 years old are underrepresented. The characteristics of our sample were largely similar to those of the general working population [42,43]. The sample has a small overrepresentation of questioners between 40 and 49 years old and of questioners working in industry. We observed an underrepresentation of respondents older than 60 years, of questioners working for a small company and of respondents working in the trade sector.

**Table 1:** Background characteristics.

Characteristic		Questioners ArboAntwoord responding to questionnaire %	All questioners ArboAntwoord (web domain data) %	**General Dutch working population**^**§**^ %
Gender	Female	47	47	44
	Male	53	53	56

Age	15-29 Years	11	17	11
	30-39 Years	23	23	23
	40-49 Years	35	23	28
	50-59 Years	28	18	25
	≥ 60 years	3	1	13
	Missing	-	19	-

Educational level	Low	23	12	22
	Intermediate	37	35	45
	High	40	34	33
	Missing	-	19	-

Occupation	OSH professional	17	16	Unknown
	Employee	67	68	
	Manager or employer	13	10	
	Other	3	5	
	Missing	-	1	

Company Size	Small	34	39	43
	Medium	38	29	37
	Large	26	24	20
	Missing	2	8	-

Sector	Agriculture and fishery	3	Unknown	1
	Industry	18		12
	Construction industry	4		5
	Trade	8		16
	Transport and communication	5		6
	Financial services	2		5
	Business services	19		17
	Public Policy/Civil Service	4		7
	Education	8		7
	Healthcare	19		16
	Culture and other services	9		8
	Missing	1		-

Internet use per week	0-10 hours	52	Unknown	Unknown
	≥ 11 hours	46		
	Missing	2		

Background characteristics of the ArboAntwoord questioners responding to the questionnaire (N = 232), of all ArboAntwoord questioners based on their web domain data (N = 460) and of the general Dutch working population.

^**§**^Data extracted from the national bureau of statistics (CBS) [42] and research centre TNO [43], both accessed August 4^th ^2011. The samples include both active and non-active work-participating individuals.

### Quality and overall satisfaction

A large majority of the questioners perceived the network as user friendly (81%) and easily accessible (75%). A slightly smaller percentage of questioners found the network easy to handle (67%). Most questioners perceived the information received as complete (69%) and applicable (70%), and almost all believed they had received the information on time (84%). Almost three quarters of the questioners were (very) satisfied with the ArboAntwoord expert network (71%), 18% percent were not satisfied or dissatisfied and 11% were (very) unsatisfied. We analysed the relationship between the six variables related to the perceived quality of the network and information and the overall satisfaction with the network facility. All six network and information variables were positively associated with the overall satisfaction in univariate analyses (p < 0.05) (Table 2). In contrast, none of the characteristics of the questioners such as age, gender, educational level or function were related to overall satisfaction.

**Table 2:** Analyses of variables that may influence questioners' overall satisfaction with the online network ArboAntwoord.

Characteristics of questioners and aspects related to perceived quality of the network and of the information received (N)		Satisfied n(%)	Not satisfied n(%)	Satisfied vs. not satisfied p
Gender	Female	70 (67)	34 (33)	
(N = 227)	Male	92 (75)	31 (25)	0.21

Age group	15-29 years	20 (77)	6 (23)	
(N = 227)	30-39 years	38 (73)	14 (27)	
	40-49 years	56 (71)	23 (29)	
	50-59 years	46 (72)	18 (28)	
	≥ 60 years	2 (33)	4 (67)	0.14

Educational level	Low	34 (67)	17 (33)	
(N = 227)	Intermediate	65 (77)	19 (23)	
	High	63 (69)	29 (31)	0.48

Occupation	OSH professional	27 (73)	10 (27)	
(N = 227)	Employee	107 (70)	45 (30)	
	Manager or employer	24 (77)	7 (23)	
	Other	4 (57)	3 (43)	0.48

Company Size	Small	56 (73)	21 (27)	
(N = 224)	Medium	60 (70)	26 (30)	
	Large	45 (74)	16 (26)	0.50

Internet use per week	0-9 hours	83 (70)	36 (30)	
(N = 227)	≥ 10 hours	79 (73)	29 (27)	0.57

User friendliness of the network	User friendly	138 (79)	36 (21)	
(N = 215)	Not user friendly	19 (46)	22 (54)	< 0.001

Network was easy to handle	Easy	115 (80)	28 (20)	
(N = 215)	Not easy	42 (58)	30 (42)	0.001

Accessibility of the network	Easily accessible	128 (80)	33 (20)	
(N = 215)	Not easily accessible	29 (54)	25 (46)	< 0.001

Completeness of the information received	Complete	128 (85)	23 (15)	
(N = 220)	Incomplete	31 (45)	38 (55)	< 0.001

Applicability of the information received	Applicable	139 (91)	14 (9)	
(N = 220)	Inapplicable	20 (30)	47 (70)	< 0.001

Timeliness of the information received	On time	141 (76)	44 (24)	
(N = 220)	Not on time	18 (51)	17 (49)	0.003

Univariate associations between questioner characteristics, perceived qualities of the network, perceived qualities of the information received and questioners' overall satisfaction with the expert network ArboAntwoord.

A multiple logistic regression analysis with backwards procedure including the six significant variables from the univariate analysis (p < 0.1) confirmed that three variables were positively associated with questioners' overall satisfaction. The Hosmer and Lemeshow test showed a significance level above 0.05, which indicates that this final predictor model fits (χ^2 ^= 1.8; p = 0.8). The applicability of the received information was most strongly associated with overall satisfaction followed by the user friendliness of the network and completeness of the information received (Table 3). The regression equation of the model (constant = -2.234) showed that a worker with an OSH question who perceived the network as user friendly and the received information as applicable and complete had a 95% likelihood of being satisfied, compared to 32% for a questioner without any of these three positive judgements.

**Table 3:** Regression model predicting questioners' overall satisfaction with the online network ArboAntwoord.

Factor†	Coefficient B	Standard Error SE	Wald X2	Odds Ratio OR (95%CI)
**Applicability of the information received**	2.8	0.4	43.8	16.0 (7.0-36.4)**

**User friendliness of the network**	1.2	0.5	6.1	3.3 (1.3-8.6)*

**Completeness of the information received**	1.1	0.4	6.7	3.0 (1.3-6.8)*

Model predicting questioners' overall satisfaction with the online expert network ArboAntwoord by the applicability and completeness of the information received and the user friendliness of the network (multiple logistic regression analysis; N = 215).

†Six significant variables (p < 0.1) from the univariate analyses were included in the multiple logistic regression analysis.

* p < 0.05

** p < 0.001

### Impact

Information increased the knowledge and understanding of 153 of 206 questioners (74%). Qualitative analyses of the data from the related open questions showed that questioners often used the information they received from ArboAntwoord experts as a foundation for good judgement, support for a decision or a second opinion. For example, one questioner wrote "I could now provide an answer to a question that management asked me to solve." Another said "Discussion issue can be checked off: good reliable information shed better light on this."

In total, 51 of 205 questioners (25%) reported that the information had or will have an impact on their work or work functioning. This percentage increased to 34% in a subgroup analysis that only included the data of the questioners who had the intention to actually apply the answer to their work situation (67% of total). Answers to the open questions illustrated that the information often had an impact beyond that of the individual questioner. For 32 of the questioners (16% of total), the information facilitated changes in working conditions at the company or department level (Table 4). For example, one respondent mentioned "Finally, the dust accumulating tapestry was removed from the office and replaced." Also, on the individual level the information sometimes helped to improve the workplace or work situation (6%), and for some questioners, the information even helped to reduce health complaints or improve work functioning (3%). Table 4 includes several illustrative remarks made by OSH questioners who perceived that the received information had an impact on their work or work functioning. A final subgroup analysis showed that the impact of the information was influenced by company size (p = 0.049). In our sample, an answer to a question from a worker from a large company impacted work or work functioning significantly more often than an answer to a question from a worker from a small or medium-sized company. Impact was not influenced by gender, age, educational level, occupation and internet use (p > 0.05).

**Table 4:** Illustrative remarks of questioners related to the impact of information on work or work functioning.

Impact information Level of impact (Description of impact)	Illustrative remarks	Questioners who perceived information had an impact on work or work functioning n (%)
Company level: Information improved working conditions in a company or department.	"The information supported my advice to the manager to buy new office chairs. We will soon test two types of chairs."	32 (16)
		
	"Now we have improved registration methods in the case of calamities or safety accidents."	
		
	"Working clothes were purchased for the entire department."	

Individual level related to work and workplace: Information improved workplace or work situation.	"I do not have to work in soldering fumes any more. I have spent a lot of time solding and inhaled the fumes all day. I did not have any local exhaust ventilation, but now the system will arrive tomorrow."	13 (6)
		
	"...now I have more moments of rest and less work pressure..."	
		
	"My work took too much of my energy. It caused me stress. With the information, my boss and I could establish adjustments in my working time and rest-breaks."	

Individual level related to work functioning and health: Information improved work functioning or reduced complaints.	"I got new glasses, tailor-made for working with the computer. I see better now, and I am less tired."	6 (3)
		
	"I worry less. The noise at work is not dangerous; it can not impair my hearing. Nevertheless, the noise can cause interference with my work and communication."	

Illustrative remarks made by OSH questioners who perceived that the received information had an impact on their work or work functioning (N = 205).

## Discussion

### General Findings

Overall, most OSH questioners perceived the network ArboAntwoord as user friendly (81%), easily accessible (75%) and easy to handle (67%). The majority judged the information received as complete (69%) and applicable (70%), and almost all questioners believed they had received this information on time (84%). Seventy-one percent were (very) satisfied with the network overall. A multiple logistic regression analysis showed that the applicability of information to the specific context of the question(er) had the highest association with the questioners' overall satisfaction. The completeness of the information received and the user friendliness of the expert network also had a positive influence on questioners' overall satisfaction. For 74% of the OSH questioners the received information increased knowledge and understanding. For 25% of the questioners, the received information changed their work, work functioning or health, i.e., for 16%, the impact of the information went beyond the individual questioner as it helped to improve working conditions in a company or department; for 6%, the provided information facilitated the improvement of an individual workplace or work situation, and for a limited number of questioners (3%), the information could improve work functioning or reduce health complaints.

### Comparison with literature

#### Quality and overall satisfaction

Although research has shown that health-related expert facilities can produce satisfied health questioners [4,29,36,37,44], studies on factors that can predict questioners' overall satisfaction in the health care setting are scarce [45,46]. In concordance with our findings, Petter and Fruhling [46] and Biemel and Hassanein [45], who both studied health-related information facilities, found that the quality of an information facility and the quality of the information it provides are associated with overall user satisfaction. Outside the field of healthcare, more information is available. An extensive literature review including 180 papers on the use and success of new information systems concluded that there is strong evidence that both the perceived quality of a new facility and the quality of the information it provides influence user satisfaction [40].

Our specific findings on the influence of user friendliness, applicability and completeness are difficult to compare to prior research because the quality of the information facility (e.g., usefulness, learnability and ease of use) and the quality of the information it provides (e.g., applicability, completeness and correctness) are to our knowledge always analysed as composed constructs. In our study, neither quality aspects were studied as composed constructs because our aim was to provide specific knowledge to help create or redesign health-related expert services and because of the poor internal consistency identified.

Our analysis of the separate variables suggests that online expert networks in the field of OSH that aim to satisfy questioners could especially focus on the applicability of information, although user friendliness of the network and completeness of information should also be addressed. In particular, experts should try to provide information that addresses not only the essential "main" question but also the context of the question(er). To accomplish this, a dialogue between questioner and expert may be important. Although the ArboAntwoord facility provides the possibility to provide feedback on a question or answer for further clarification, illustration or information, this feature is not often used. It is possible that the dialogue has to be stimulated more or that other types of dialogue features have to be added, such as a chat box with the experts. Combining network features with popular new mobile or computer application software like WhatsApp Messenger or Skype may also help stimulate the dialogue between questioner and expert. Both the construct of applicability and the construct of completeness require further exploration to determine what makes information applicable and complete to OSH questioners. Finally, usability testing before launching a new expert network is essential for addressing user friendliness [31,47].

#### Impact

While we could not find specific studies on the impact of expert networks or services in the field of OSH, many studies and reviews in other fields of healthcare have shown that (online) health information and advice can have an impact on patients, health consumers, professionals and policy makers. Online information and decision support systems have been shown to impact the knowledge and understanding of its users, several health-related behaviours and treatment decisions [8,48-52]. Similarly, research has shown that internet social networking, support groups and communities have influenced knowledge, decision making, feelings of social support, emotional well-being and health-related behavioural actions in patients with all kinds of illnesses [53-57].

In our study, the information provided by the experts had an impact on the questioners' OSH situation. The information was more likely to increase the questioners' knowledge and understanding than to directly impact their work, work functioning or health. As the majority of questioners had the intention to actually apply the information to their work situation, this finding can be partially explained by the fact it generally takes more time than 90 days to realise an effect on these outcomes. Another explanation might be that a number of questions did not require any immediate action to change behaviour or work. For example, the question "can computer work, for 8 hours a day, 5 days a week cause my low-back pain?" may worry people but often does not need direct action. Questions asked in ArboAntwoord were generally not very urgent in the sense of requiring acute care. Especially in the context of OSH questions with a relatively low urgency, another cause might explain the difficulty of accomplishing behavioural change. In the theory of planned behaviour [58] and the ASE-Model [59], behavioural intention is influenced by attitude, social influence and (self) efficacy. These factors may either facilitate or deter OSH questioners to take action. The theory of diffusion of innovations may also provide a useful perspective. In this theory, a behavioural change is preceded by several essential consecutive behavioural states and steps: knowledge, persuasion, decision, implementation and confirmation [60]. Likely, not all questioners are reaching all of these behavioural states, which may hinder them taking action. It might be interesting to further explore the factors that facilitate or hinder OSH questioners to take action on the information or advice. We did found that an answer to a question from a worker from a large company impacted work or work functioning significantly more often than an answer to a question from a worker from a small or medium-sized company. Possibly, large companies have a better internal OSH infrastructure, such as in company OSH professionals or human resource managers who can further realize changes in OSH.

In contrast to our expectations, the information provided by experts more often had an impact on working conditions at the company level than on the individual level. One might expect that managers or OSH professionals have more influence on OSH at the company or department level than individual workers. Our findings did not support that idea. It is possible that workers used the received information to try to change colleagues' behaviour or work directly or by contacting the work council, a safety engineer or human resources manager, people within a company who have the capability to introduce the issue in a broader company context.

Our study demonstrates that an online expert network can be a useful new strategy for improving both OSH knowledge and working conditions. Nevertheless, especially the perceived quality of the information and the impact on OSH requires further study. Future studies could compare the perceived quality and impact of online expert networks and other expert facilities with that of common information facilities, such as informational websites. Possibly, expert networks could be embedded within existing information sources, such as high-quality informational websites (e.g., when the questioner is not able to find an answer on the website, he can be transferred to an expert). The choice to study an expert network related to a specific OSH topic, e.g., OSH law and regulation, chemical risks or return to work, could facilitate operationalisation and comparison of the perceived quality and impact.

### Limitations

Several methodological limitations should be noted. First, our study's cross-sectional design constitutes a limitation. It would have been superior to use a longitudinal study to predict questioners' satisfaction, and a comparative study, utilising another (common) information facility, to evaluate impact. Furthermore, the response rate of 45% and the limited number of 205 respondents may have resulted in some selection bias. Possibly, satisfied questioners for whom the information changed their knowledge, working conditions or work functioning might be overrepresented in our sample. Conversely, dissatisfied questioners might also have been motivated to fill in the questionnaire. Because of a potential selection bias and a lack of detailed information on the non-responders, caution is required when generalising our findings to working populations using online OSH information. That fact that our questionnaire was not extensively pre-tested and validated constitutes another limitation. In particular, the findings on impact require further consideration. Even when the provided information aided in removing or improving potential OSH risks, the actual impact can often only be expected after a longer period of time. Second, it may have been better to include specific impact related outcomes. As the OSH questions spanned a variety of topics, natures and purposes, we only asked three non-specific questions to explore the impact. Not specifically asking the questioners about the impact of the information on health complaints could partially explain why only 3% of the questioners' information impacted this outcome. Additional qualitative study designs, e.g., by interviewing questioners about the impact of information received, might have revealed more complete data on the impact of information on OSH.

## Conclusions

A free-of-charge online expert network can be a useful additional strategy for providing applicable, complete and timely information to OSH questioners that may help improve safety and health at work. Our findings show that both network quality and perceived information quality are associated with questioners' overall satisfaction with an online expert network. When aiming for satisfied questioners, network experts should especially focus on the applicability of the information to the context of the questioner, as this aspect was associated most strongly with questioners' overall satisfaction. Online expert networks in the field of OSH have the potential to positively impact OSH both with regard to knowledge and working conditions at both the individual level and company level.

## Competing interests

The authors declare that they have no competing interests.

## Authors' contributions

MR, CH, FD and AL designed the study. MR and CH planned the analysis and collected data. MR analysed data. MR, CH, FD and AL wrote the paper. All authors read and approved the final manuscript.

## Pre-publication history

The pre-publication history for this paper can be accessed here:

http://www.biomedcentral.com/1472-6947/11/72/prepub
